# Trends in school‐neighbourhood inequalities and youth obesity: Repeated cross‐sectional analyses of the public schools in the state of California

**DOI:** 10.1111/ijpo.12991

**Published:** 2022-12-14

**Authors:** Mika Matsuzaki, Emma V. Sanchez‐Vaznaugh, Kelsey Alexovitz, Maria E. Acosta, Brisa N. Sánchez

**Affiliations:** ^1^ Department of International Health Johns Hopkins Bloomberg School of Public Health Baltimore Maryland USA; ^2^ Center for Human Nutrition Johns Hopkins Bloomberg School of Public Health Baltimore Maryland USA; ^3^ Department of Public Health San Francisco State University San Francisco California USA; ^4^ Health Equity Institute San Francisco State University San Francisco California USA; ^5^ Center for Health Equity University of California San Francisco California USA; ^6^ Department of Epidemiology and Biostatistics Drexel Dornsife School of Public Health Philadelphia Pennsylvania USA

**Keywords:** gender, neighbourhood inequality, race/ethnicity, school, urbanicity, youth obesity

## Abstract

**Background:**

It is currently unknown whether the relationship between affluence of school neighbourhoods and prevalence of youth overweight/obesity is uniform across demographic subgroups and areal context in the United States.

**Methods:**

We examined association between school‐neighbourhood income tertiles and school‐level overweight/obesity (OVOB) prevalence, using data on body mass index of fifth, seventh, and nineth graders who attended public schools in California in 2001 and 2010 (*n* = 1 584 768), using multiple logistic regression models.

**Results:**

Overall, OVOB prevalence was higher in lower‐income school neighbourhoods, with a steeper income‐OVOB gradient for girls. Among boys, the gradient became steeper in 2010 than 2000. Among Asian and White girls, the negative gradients were steepest in rural areas. For African–American students in all areas and Latino boys in rural areas, there was less clear evidence of inverse income‐OVOB gradients. Addition of fast‐food restaurant availability to the models did not change the observed inverse school‐neighbourhood income–obesity gradients.

**Conclusions:**

The findings suggest the needs to investigate reasons for this variability with consideration to combinations of sociodemographic, economic, and environmental risk factors that may contribute to disparities in childhood obesity.

## 1 INTRODUCTION

In the United States (U.S.), one in three children are classified as either overweight or obese.^1^ Children from low‐income households and neighbourhoods and from racial/ethnic minority populations generally have higher overweight and obesity prevalence.^1^, ^2^, ^3^, ^4^ However, this relationship between income and obesity may be variable across sociodemographic characteristics. Studies have shown wider gaps in youth obesity prevalence by household or neighbourhood income levels for girls than boys while the income–obesity gradient is less clear for some racial/ethnic groups such as African Americans.^2^, ^5^, ^6^, ^7^ This variability in income–obesity relationships requires attention to sociodemographic characteristics to understanding multifactorial causes of youth obesity.

Children in the U.S. spent an average of 6.67 h of their waking hours in and around schools in 2007–2008.^8^ Some students go off campus during those hours to purchase foods and drinks for consumption. Additionally, a study in San Francisco found that in 2013, about a quarter of sugar‐sweetened beverages (SSBs) consumed at school were purchased during commute and students who purchased SSBs during commute were more likely to consume SSBs at school.^9^ Schools in low‐income neighbourhoods may face a plethora of challenges in promoting healthy lifestyles among schoolchildren. For instance, unsafe neighbourhood environment and scarce green space may also contribute to high burden of childhood obesity in low‐income populations.^10^ Poor quality of neighbourhood food environment is one potential factor that can explain—and may become a point of interventions to mitigate—disproportionately high prevalence of overweight/obesity in socioeconomically disadvantaged communities. Thus, we hypothesize that unhealthy food environments near schools may partially explain the association between school‐neighbourhood income patterns and childhood obesity prevalence.^11^, ^12^

Despite the potential differences in risk factors for youth obesity by school‐neighbourhood characteristics, a few studies have shown how economic inequalities in school neighbourhoods may relate to youth obesity. Hence, using comprehensive data on public schools and students in California in 2000 and 2010, we investigated: (1) youth overweight/obesity prevalence by school‐neighbourhood income level by subgroups of gender, race/ethnicity, and levels of urbanicity in which schools are located, (2) whether disparities in OVOB prevalence between low‐ and high‐income school neighbourhoods widened over time, and (3) whether—and to what extent—availability of unhealthy food outlets near schools explained the association between school‐neighbourhood income and OVOB prevalence.

## 2 METHODS

Our study focused on assessment of school‐neighbourhood income levels and school‐level OVOB prevalence derived from student‐level data. In the following sections, we refer to the areas where the schools are located as *neighbourhoods* and the median income levels of the residents in those areas as *income*, respectively.

### 2.1 Data Sources

For estimating the school‐level OVOB prevalence, child‐level data on age, gender, grade, race/ethnicity, fitness level, and BMI were obtained from the Fitnessgram test administered annually to all public schoolchildren in the state.^13^ Children with Fitnessgram data who attended primary, middle, and high schools that were open in both 2001 and 2010 and had student enrolment data were included in this analysis.

School‐level data were obtained from multiple sources. Publicly available school‐level data on enrolment and demographics were obtained from the California Department of Education website.^14^ Census tract‐level annual median household income was obtained from the 2000 and 2010 Census to determine levels of socioeconomic resources within the school's neighbourhoods, defined as the census tracts in which schools were located. Statewide fast‐food restaurant (FFR) locations were obtained from the National Establishment Time Series (NETS) database for 2001 and 2010.^15^ Proprietary Nielsen PRIZM urbanization data from 2013 were used to classify school locations according to the level of urbanization in the area where schools were located.^16^

### 2.2 Measures

Body mass index (BMI) was calculated by dividing weight in kilograms (kg) by height in meters squared (m^2^). BMI z‐scores for each child were derived by cross‐referencing the child's BMI to the Centre for Disease Control's 2000 age‐ and gender‐specific BMI growth charts. Students were classified as normal/underweight or overweight/obese based on whether the child was below or above the BMIz‐score value corresponding to the 85th percentile of the CDC growth chart.^17^

Children's fitness status was based on whether they exceeded, met, or did not meet the Cooper Institute's guidelines for the time to run a mile for children of a given age and gender. If students were missing mile‐run data, we used data from the PACER test instead, by converting the number of laps in the PACER test into minutes needed to run a mile using a published equivalency chart.^18^

The schools' census tract membership for 2001 and 2010 was determined for each school based on its geocoded address. We examined these data to compare the findings to our prior work during the same period assessing the association between school‐neighbourhood income and fast‐food outlet availability. Data on the median household income of the residents at the census tract level was linked to each school's census tracts, as a measure of the school's neighbourhood socioeconomic environment. Median household income (hereafter income) was categorized into tertiles. Based on the Nielsen PRIZM data urbanization data, schools were classified into one of four categories depending on the urbanicity classification for the school's census tract: urban, second city, suburban, rural. Urban areas are those with high population density and are within major metropolitan areas. Second city areas are moderately dense population centres in smaller cities or large towns. Suburban areas are moderately dense areas near urban or second city core areas. The rural category refers to small towns and other low density areas that are either part of the urban fringe or standalone low population density areas.^16^ The proportion of students eligible for Free or Reduced Price Meals (FRPM) for each school for each year was calculated as a proxy for school‐level socioeconomic characteristics.^19^

FFR availability was defined as the number of both chain and non‐chain fast‐food restaurants within each school's 1/4‐, 1/2‐, 3/4‐ and 1‐mile network buffer. Buffers of different sizes where used given that there is no consensus on the most appropriate spatial scale within which to measure child‐relevant food environment features. FFR chains were identified as those that appear on the list of fast‐food eating places regardless of Standard Industry Classification code. Non‐chain FFR were identified with SIC codes for food outlets that specialize in low preparation time foods that are eaten cafeteria style (no waiter service) or takeaway.

### 2.3 Statistical analysis

Descriptive statistics were calculated separately for students and schools overall, and by school‐neighbourhood income tertile. Crude student‐level prevalence of overweight/obesity was calculated by neighbourhood income tertile and year, for girls and boys separately, as well as by race/ethnicity, urbanicity, and by urbanicity within race/ethnicity strata.

A series of multilevel logistic regressions were fitted to systematically investigate adjusted neighbourhood income‐OVOB patterns and examine whether the food environment partially explained them. We examined this association by urbanicity of school locations, racial/ethnic composition of the study body, and gender of students. The school locality (i.e., urbanicity) is associated with built environment by definition (e.g., more or less retail shops), which includes food environment. This leads to differences in the types of built environments students are exposed to. For instance, students attending schools located in affluent areas in urban settings have exposures to different built environments from students in schools in affluent rural areas. Such differences may influence the impact of environmental differences by school‐neighbourhood income on schoolchildren's diet and weight status. Additionally, commuting methods may also vary by urbanicity. We were also how they may or may not shape obesity prevalence across children of different racial/ethnic backgrounds as well as socioeconomic disparities in obesity within racial/ethnic groups. Additionally, we stratified the analyses by race/ethnicity because previous research has shown racial/ethnic differences in the association between income and obesity. Thus, those differences would be masked if we combined all racial/ethnic groups in the analyses.

The models used child‐level overweight/obese status as the dependent variable, with tertiles of income as the primary independent variable. Interaction terms between income tertiles and academic year (2010 vs 2001) were included to examine if the pattern of association in 2010 changed from that of a decade earlier. All models were adjusted for student‐level characteristics (age and fitness status) and school‐level characteristics (school classification and school size). The models accounted for the similarity (clustering) of children's overweight/obesity status within schools and school districts by using random intercepts and slopes of academic year for school districts, and schools within school districts.

Models were constructed separately by child gender, given well‐established gender‐differences in children's obesity related to gender‐specific pubertal tempo and concomitant changes in adiposity. The gender‐stratified models were fitted: (a) among children of all race/ethnic groups combined to estimate overall income‐OVOB patterns, while adjusting for child race/ethnicity in addition to the above listed covariates; (b) stratified by child‐level race/ethnicity, to more strongly control for it given the high correlation between income and race/ethnicity. Additionally, models were (c) stratified by the urbanicity levels in which the schools were located, to more strongly control for urbanicity given its association with both income and obesity. The final set of models were stratified by (d) urbanicity and child‐level race/ethnicity combined, to further isolate the income‐OVOB patterns, given the correlations among these two factors.

All models were subsequently adjusted for FFR availability measures to examine the extent to which the food environment near schools explained (i.e., attenuated) any observed income‐OVOB patterns. Food environment measures consisted of the number of FFR and/or convenience stores in the school neighbourhood, defined as network buffers of different sizes. We considered models where food environment measures were treated as time‐varying predictors, as well as where the measures were disaggregated into the count of outlets in 2001 and the change in the count of outlets from 2001 to 2010. The counts of food outlets were initially modelled using a linear term. However, given prior research demonstrating lack of linearity in the food‐environment‐OVOB association, lack of linearity was explored using splines with two degrees of freedom as well as quadratic transformations. The use of several specifications for the food environment measures enabled us to minimize the possibility that lack of explanation of the income‐OVOB association by food environment was due to poor modelling of the available food environment metrics. We stratified these models by grade but did not see evidence for differences in the effects of FFR availability by grade. Therefore, we combined all grades in the final models.

Since power to detect interactions is lower than for main effects, we used the 0.10 level of significance to screen for significant income‐by‐year interactions, while using the typical 0.05 level for main effects of income levels on overweight/obesity. Adjusted odds ratios were calculated through exponentiated regression coefficients.

## 3 RESULTS

The overall youth overweight/obesity prevalence in this study population was 37%. The prevalence was highest among the students attending schools in the lowest income tertile neighbourhoods (43.7%) and lowest among those in the highest income tertile (29.9%) (Table 1). There were differences in the racial/ethnic composition across school‐neighbourhood income tertiles, where Latino students were the largest group within the lowest and middle income school neighbourhoods (68.1% and 55.3% respectively), while White students were the majority racial/ethnic group for the schools within the highest income neighbourhoods. Nearly, forty percent of the students from the lowest income neighbourhoods were classified in the group who needs improvement in their fitness levels (39.2%), much higher than children attending schools in the highest income neighbourhoods (25.3%).

**TABLE 1:** Characteristics of students by school‐neighbourhood income tertiles

	Lowest	Middle	Highest	All
Characteristics	*N* = 459 596	*N* = 540 920	*N* = 584 252	*N* = 1 584 768
Overweight/obese, %	43.7	38.9	29.9	37
*Race/ethnicity, %*				
African American	9	7.8	6.2	7.6
Asian	3.8	6.4	14	8.5
Latino	68.1	55.3	29.5	49.5
White	19	30.5	50.3	34.5
*Gender, %*				
Girls	49.2	49.1	48.9	49
Boys	50.8	50.9	51.1	51
*Fitness status, %*				
Needs improvement	39.2	34.4	25.3	32.5
Meets standards	41·4	44.7	47.9	44.9
Exceeds standard	13.7	16	22.5	17.7
Missing	5.6	4.9	4.3	4.9
*Age (Years), %*				
9	<0.1	<0.1	<0.1	<0.1
10	22.1	18.7	18.2	19.5
11	15.8	13.8	14.5	14.6
12	18.9	19.8	20.2	19.7
13	13.7	14.7	15.4	14.7
14	15.4	17.6	17.3	16.9
15	12.2	13.8	13.6	13.3
16	1.7	1.4	0.7	1.2
17	0.2	0.1	<0.1	0.1
*Grade, %*				
Grade 5	39.0	33.3	33.2	34.9
Grade 7	32.5	34.6	35.6	34.4
Grade 9	28.5	32.1	31.2	30.7

Among the 5127 schools included in this study, schools in urban areas make up the highest percentage in lowest and middle income tertiles while schools in sub‐urban areas make up the highest percentage of schools in the highest income tertile (Table 2). There were fewer FFR available within 0.75 miles from schools located in the highest tertiles of income in both 2001 and 2010.

**TABLE 2:** Characteristics of schools by the income tertiles of the neighbourhoods in which they are located

Characteristics of schools	Lowest	Middle	Highest	All
Total *n*	1475	1740	1912	5127
*Urbanicity, % (n)*				
Rural	21.3 (314)	14 (244)	7.8 (150)	13.8 (708)
Second city	19.5 (287)	21·1 (368)	14.4 (276)	18.2 (931)
Suburban	7.1 (105)	15.2 (265)	40 (765)	22.1 (1135)
Urban	52.1 (769)	49.6 (863)	37.7 (721)	45.9 (2353)
*FFR availability (0·75 Mile), mean (SD)*				
2001	3.0 (3·8)	2.5 (2·9)	1.9 (2·6)	2.4 (3·1)
2010	3.2 (4·0)	2.6 (3·0)	1.8 (2·5)	2.5 (3·2)
*School classification, % (n)*				
Elementary school	69.3 (1022)	68 (1183)	69.2 (1323)	68.8 (3528)
K‐12	1.4 (20)	1.1 (19)	0.7 (13)	1 (52)
Middle school	15.7 (232)	17.4 (303)	17.4 (333)	16.9 (868)
High school	13.6 (201)	13.5 (235)	12.7 (243)	13.2 (679)
*Total enrolment, mean (SD)*				
2001	934 (690)	870 (625)	846 (576)	880 (628)
2010	784 (598)	789 (612)	819 (588)	799 (599)

Abbreviations: FFR, fast food restaurant; SD, standard deviation.

**FIGURE 1:**
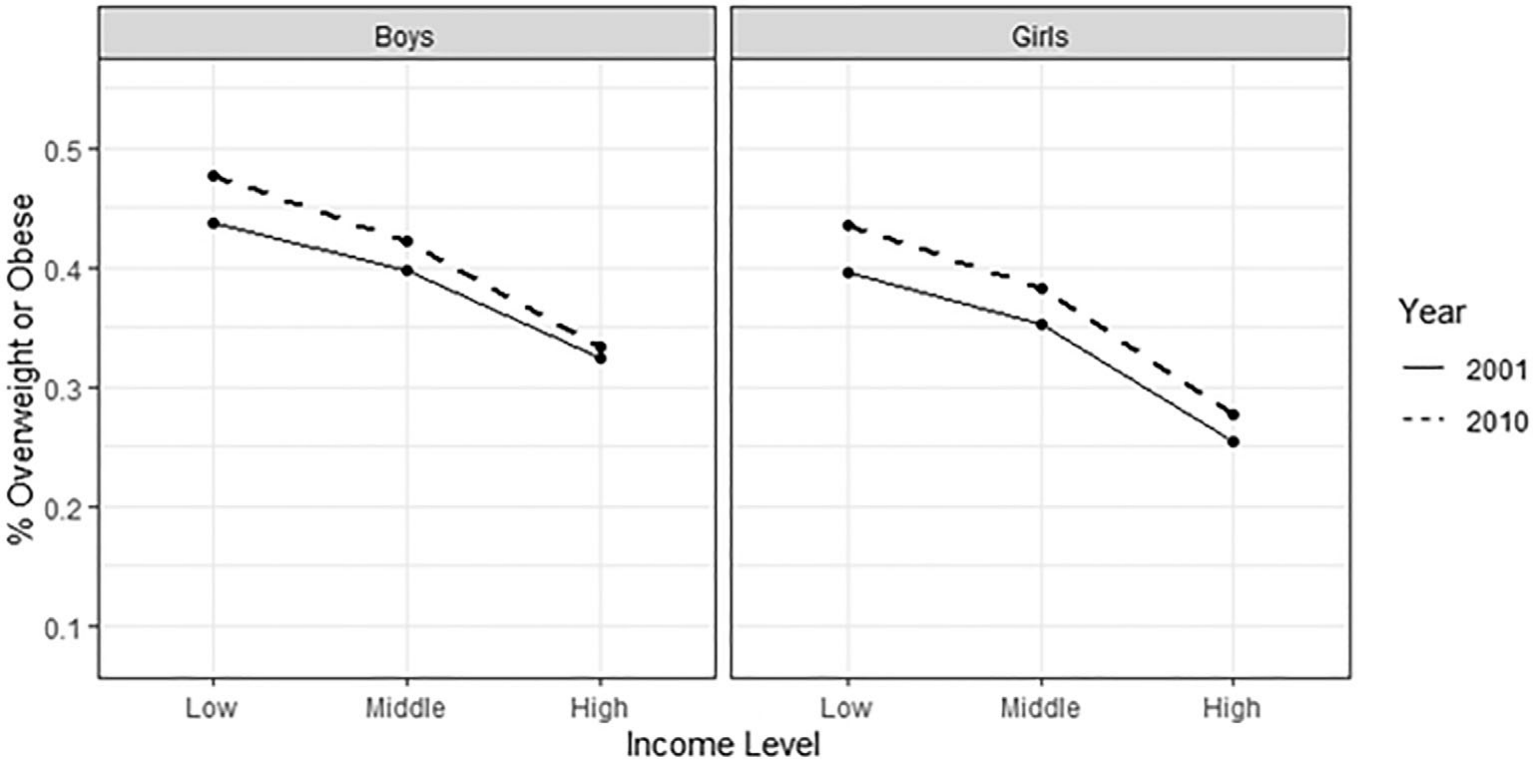
Proportion of schoolchildren in fifth, seventh, and nineth grades with overweight/obesity by school‐neighbourhood income tertiles stratified by gender

First, we compared the association between school‐neighbourhood income tertiles and OVOB by gender. For both girls and boys, we saw higher OVOB prevalence in lower‐income school neighbourhoods (Figure 1). The prevalence of OVOB increased between 2000 and 2010 for all neighbourhood income strata. However, this income‐OVOB gradient was steeper in 2010 in comparison to 2001 for boys (high‐income tertile by year interaction *p*‐value = 0.0323).

### 3.1 Race/ethnicity stratification

When the models were further stratified by child‐level race/ethnicity, the inverse association persisted for all subgroups, except African–American boys (Appendix Figure S2). The test for the interaction between income tertiles and year showed a steeper income‐OVOB gradient in 2010 than 2000 for Asian boys only.

### 3.2 Urbanicity stratification

Next, we examined the association between income levels and overweight/obesity separately for boys and girls attending schools in urban, second city, suburban, or rural area. In the crude analyses, across all areas, we saw the same pattern of higher OVOB prevalence among children attending schools in lower‐income neighbourhoods in both 2001 and 2010 (Appendix Figure S3). When we tested for the change in the steepness of the income‐OVOB gradients over time, we did not see clear evidence of increase over time for any of the urbanicity groups.

### 3.3 Joint stratification by race/ethnicity and urbanicity

When jointly stratifying by child‐level race/ethnicity and urbanicity of school locations, the income‐OVOB patterns did not change significantly between 2001 and 2010 within each strata. Thus, the interaction terms between income and time were removed. In these jointly stratified models (Appendix Figure S4), we did not find evidence of heterogeneity in the income‐OVOB gradient by urbanicity of school locations, except for Asian boys (*p* < 0.01 for the interaction of income and urbanicity) and Asian girls (*p* = 0.01), and to a lesser degree among African–American girls (*p* = 0.05) and weaker evidence among White girls (*p* = 0.10). Among White and Asian girls, the gradients were steepest in rural areas, whereas for African–American girls, the gradients were only apparent in urban areas. Among Latino boys in rural areas, we did not see evidence of inverse income‐OVOB gradients/.

Lastly, we assessed whether the availability of fast‐food restaurants near schools wholly or partially explained the association between school‐neighbourhood income patterns and OVOB, but adjusting for FFR did not attenuate this association. After adjusting for FFR, all income‐OVOB coefficients remained within 5% of the coefficients prior to this adjustment (data not shown). This lack of evidence of mediation by FFR was seen in both overall and stratified models. Additional approaches to model the food environment metrics (e.g., time varying; including convenience stores) did not result in the attenuation of the effects of income groups on overweight/obesity.

## 4 DISCUSSION

The current study found general patterns of inverse association between school‐neighbourhood income and youth overweight/obesity for most of the racial/ethnic groups and urbanicity levels of school locations. However, this pattern was less clear for African–American students. For Asian and White girls, the gradients were steepest in rural area. The income‐OVOB gradient became steeper in 2010 in comparison to 2000 for boys. Contrary to our hypothesis, the inverse income–obesity association was unexplained by the availability of fast‐food restaurants near schools.

### 4.1 Comparison to previous work

Low‐income neighbourhoods experience higher burden of childhood obesity.^5^, ^20^ These neighbourhoods, by definition, consist of a large number of low‐income households and often suffer from higher exposure to obesogenic environments, such as greater concentrations of unhealthy food outlets and lack of safe environments for physical activity.^12^, ^21^, ^22^ Unlike previous work that largely examined household and residential neighbourhood level income, the current study focused on inequalities in school neighbourhoods as a large proportion of youth spends substantial time of their active hours in or near schools and may experience exposure to various environmental risk factors for obesity.^23^ Our study provides unique perspectives on the association between school‐neighbourhood income and childhood obesity by examining this association by gender and race/ethnicity of the youth and urbanicity of school locations.

There were generally inverse associations between school‐neighbourhood income levels and youth overweight/obesity prevalence for most of the subgroups. This neighbourhood income‐OVOB gradient was steeper for girls, in line with the findings from a previous U.S. national study.^24^ One study found that boys in low‐income neighbourhoods in urban areas spend more money per purchase on foods and beverages during commute, suggesting distinct interactions with food and/or physical environments by gender.^25^ It is also important to note that in this study population, we saw evidence of widening disparities in youth obesity by school‐neighbourhood income for boys.

When further stratified by race/ethnicity, the inverse association generally persisted although unlike other subgroups it was not apparent among African–American boys. Previous studies have shown similar findings of weaker evidence for family income‐child obesity among African Americans.^26^ Previous work with MyFitnessPal data has found that in zip codes with predominantly Black populations, high incomes were associated with lower healthful food consumption, higher fast food consumption, and higher likelihood of being overweight or obese.^27^ This weaker protective effects from individual or environmental wealth require further investigation including the impact of social barriers through structural racism that African Americans across socioeconomic strata experience.^28^

We examined the urbanicity of school location as there may be distinct challenges facing children attending schools in more or less urbanized areas, regardless of neighbourhood income levels. For instance, a study has shown actively commuting was more common among boys, Latinos, those from lower‐income families, those attending public school, those living in urban areas, and those living closer to school.^29^ The evidence of a negative income‐OVOB gradient was not clear for Asian boys living in urban areas and second cities. One possible explanation is that food purchasing and dietary behaviours may be similar among Asian boys in more urbanized areas regardless of the school‐neighbourhood income levels. In rural or sub‐urban areas, there may be sociocultural differences among Asian children across the economic strata that lead to distinct dietary behaviours.

In California, previous research showed higher availability of unhealthy food outlets was observed in less affluent school neighbourhoods and this income–obesity gradient increased over time for schools with high proportions of racial/ethnic minority students.^30^ The Early Childhood Longitudinal Study also showed higher BMI among children who experienced an increase of convenience stores in school neighbourhoods, with large associations seen among girls and urban schoolchildren.^31^ In the current study, adjustment for fast‐food restaurant availability near schools did not explain the income‐OVOB association for any of the subgroups. These findings present opportunities to contemplate potential reasons. First, the impact of food environments may also require more granular analyses than our dataset could afford, including additional measures of quantity and quality of foods and drinks, prices and sales across different income neighbourhoods. A study in LA showed higher availability of fast‐food restaurants in lower‐income neighbourhoods in more commercial areas but this association was not seen in less commercial areas.^32^ This suggests that even within the same levels of urbanicity, other environmental factors may play important roles in shaping children's diet. Children in wealthier school neighbourhoods may also have more pocket money to buy and consume unhealthy foods near schools. In addition, given that school‐neighbourhood income levels also act as a proxy for household‐level income, the consistent inverse association we saw in most of the subgroups in this study population may represent greater exposure to obesogenic environments at home and in other settings outside of schools among children from low‐income households and communities.

Future research is needed to incorporate factors other than fast‐food outlets, such as other types of stores that sell caloric dense foods and sugary beverages such as pharmacies, and school‐level factors such as open campus policies (i.e., students can leave campuses during the school hours), neighbourhood safety, and physical activity environments.

### 4.2 Public health implications

These findings suggest that programs and policies to reduce socioeconomic disparities in childhood obesity should consider race/ethnicity and the urbanicity where schools are located. For example, to reduce disparities, targeted interventions may focus on youth who attend schools located in low‐income areas. While the neighbourhood income levels were generally inversely associated with childhood obesity, variations by contextual factors suggest that some areas may need to consider other factors such as resources and opportunities for physical activity. For schools that saw weaker evidence of the association between neighbourhood income and childhood obesity (e.g., schools with majority African–American students), factors within each context that may not be directly associated with neighbourhood income patterns, such as cultural and social norms around diet and physical activities, need to be considered in the development of future interventions and policies.

## 5 STRENGTH AND LIMITATIONS

The study population was from California, the most populous and one of the most diverse states in the U.S. This diversity and the size of the dataset allowed us to explore variability in the association between neighbourhood income and youth overweight/obesity across a number of demographic and socioeconomic factors. However, we lacked information on household or residential area income data, which limited our analyses to factors that affect part of children's daily lives. Nonetheless, given that commuting and school activities occupy a large portion of children's awake hours, the current study provides key information for identifying subgroups that may need interventions beyond economic equity and for developing future research and policy strategies to provide tailored interventions to improve diet and weight status among youth from socially disadvantaged populations.

## 6 CONCLUSION

Our study highlights that the inverse association between school‐neighbourhood income levels and youth overweight/obesity prevalence is present among the majority of population subgroups defined by gender and race/ethnicity. The gradient was steeper for girls although boys saw an increase in steepness of the gradient over time. For Asian children, the steepness of the gradients varied by urbanicity level of the school location. Availability of fast‐food restaurants did not explain the association between the school‐neighbourhood income inequalities and youth overweight/obesity. Future studies should explore other environmental risk factors such as physical activity environment and disparities in resources to promote healthy lifestyles.

## CONFLICT OF INTEREST

The authors declare no conflict of interest.

## Supporting information


**Figure S2:** Proportion of schoolchildren in fifth, seventh, and nineth grades with overweight/obesity by school‐neighbourhood income tertiles stratified by gender and race/ethnicity
**Figure S3:** Proportion of schoolchildren in fifth, seventh, and nineth grades with overweight/obesity by school‐neighbourhood income tertiles stratified by gender and urbanicity of school location
**Figure S4:** Adjusted odds ratios of being overweight/obese by school‐neighbourhood income tertiles stratified by gender, race/ethnicity, and urbanicity of school location
