# Supplementary material for: Trends in school‐neighbourhood inequalities and youth obesity: Repeated cross‐sectional analyses of the public schools in the state of California
Source: Pediatr Obes. 2022 Dec 14;18(3):e12991. doi: 10.1111/ijpo.12991 (PMC10078445; doi:10.1111/ijpo.12991)

SUPPLEMENTAL MATERIAL

Figure 2: Proportions of schoolchildren in 5th, 7th, and 9th grades with overweight/obesity by school-neighborhood income tertiles stratified by gender and race/ethnicity.

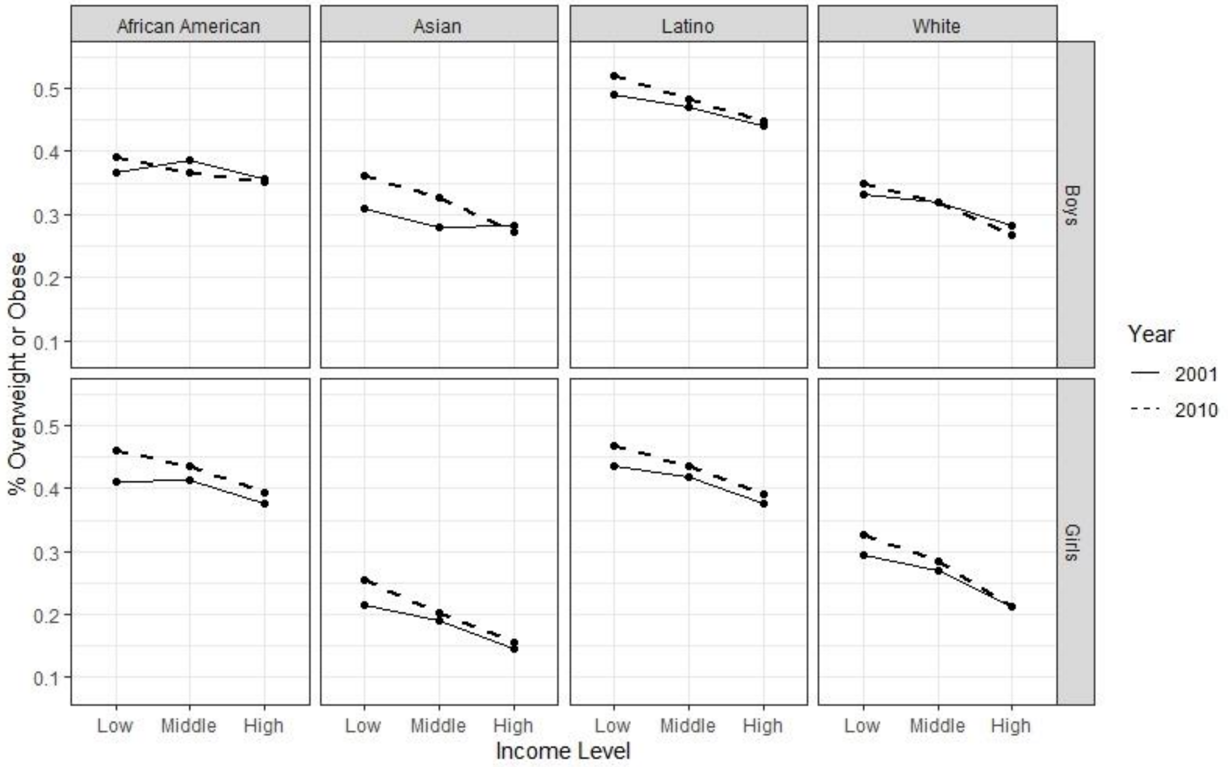

Figure 3: Proportions of schoolchildren in 5th, 7th, and 9th grades with overweight/obesity by school-neighborhood income tertiles stratified by gender and urbanicity of school location.

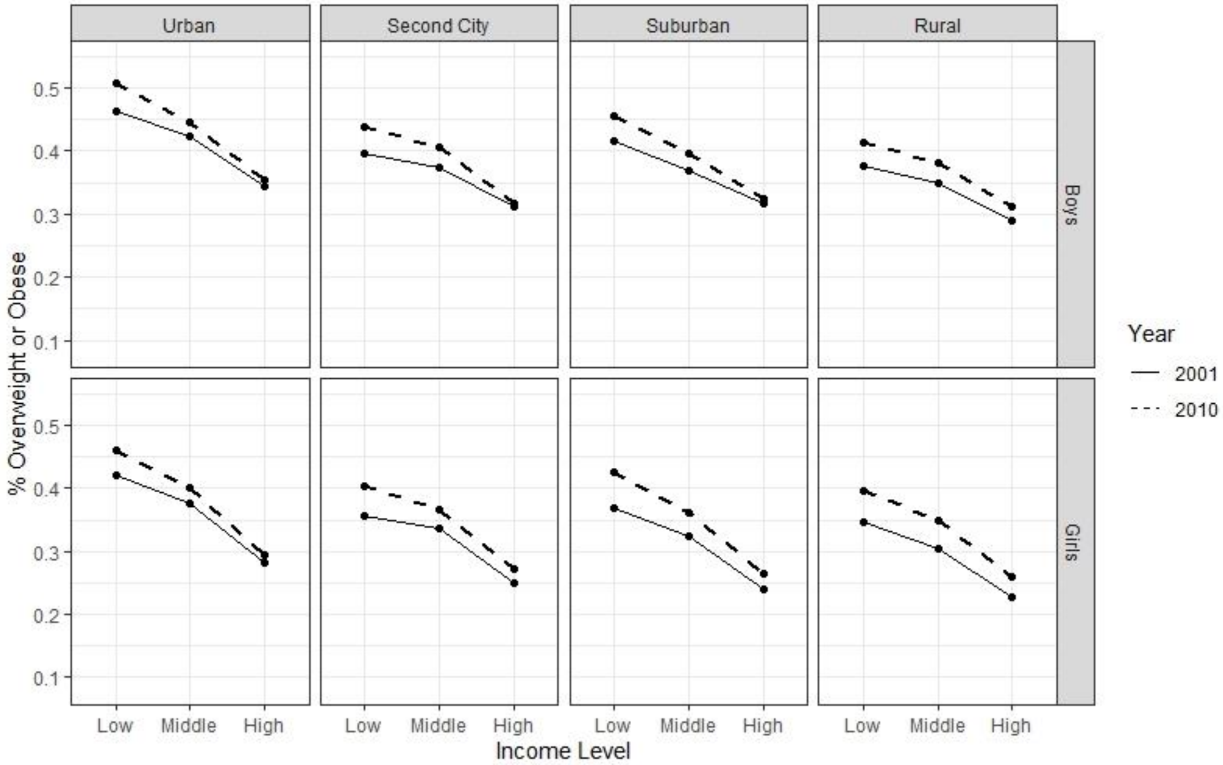

Figure 4: Adjusted odds ratios of being overweight/obese among schoolchildren in 5th, 7th, and 9th grades by school-neighborhood income tertiles stratified by gender, race/ethnicity, and urbanicity of school location.

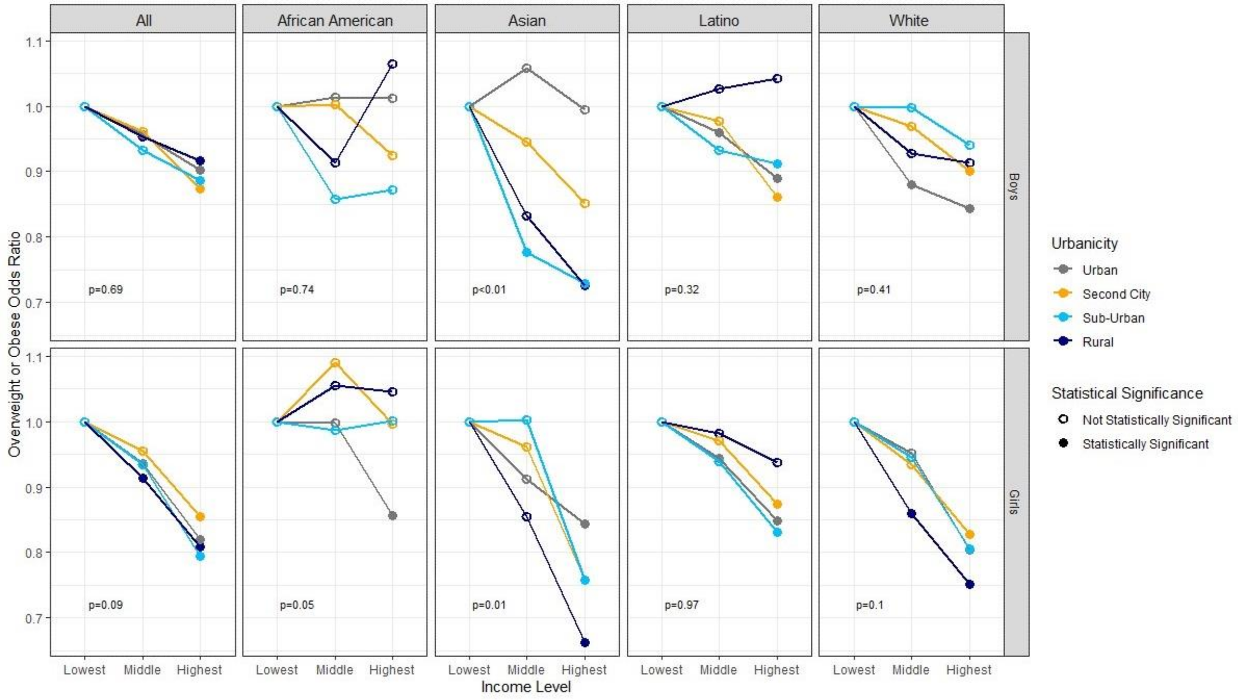

Supplement: Supplementary file 1 — Figure S2: Proportion of schoolchildren in fifth, seventh, and nineth grades with overweight/obesity by school‐neighbourhood income tertiles stratified by gender and race/ethnicity Figure S3: Proportion of schoolchildren in fifth, seventh, and nineth grades with overweight/obesity by school‐neighbourhood income tertiles stratified by gender and urbanicity of school location Figure S4: Adjusted odds ratios of being overweight/obese by school‐neighbourhood income tertiles stratified by gender, race/ethnicity, and urbanicity of school location [file IJPO-18-0-s001.pdf]
